# A Novel Computational Method for the Identification of Potential miRNA-Disease Association Based on Symmetric Non-negative Matrix Factorization and Kronecker Regularized Least Square

**DOI:** 10.3389/fgene.2018.00324

**Published:** 2018-08-21

**Authors:** Yan Zhao, Xing Chen, Jun Yin

**Affiliations:** School of Information and Control Engineering, China University of Mining and Technology, Xuzhou, China

**Keywords:** microRNA, disease, association prediction, matrix factorization, Kronecker regularized least square

## Abstract

Increasing evidence has indicated that microRNAs (miRNAs) are associated with numerous human diseases. Studying the associations between miRNAs and diseases contributes to the exploration of effective diagnostic and treatment approaches for diseases. Unfortunately, the use of biological experiments to reveal the potential associations between miRNAs and diseases is time consuming and costly. Therefore, it is very necessary to use simple and efficient calculation models to predict potential disease-related miRNAs. Considering the limitations of other previous methods, we proposed a novel computational model of Symmetric Nonnegative Matrix Factorization for MiRNA-Disease Association prediction (SNMFMDA) to reveal the relation of miRNA-disease pairs. SNMFMDA could be applied to predict miRNAs associated with new diseases. Compared to the direct use of the integrated similarity in previous computational models, the integrated similarity need to be interpolated by symmetric non-negative matrix factorization (SymNMF) before application in SNMFMDA, and the relevant probability of disease-miRNA was obtained mainly through Kronecker regularized least square (KronRLS) method in our model. What's more, the AUC of global leave-one-out cross validation (LOOCV) reached 0.9007, and the AUC based on local LOOCV was 0.8426. Besides, the mean and the standard deviation of AUCs achieved 0.8830 and 0.0017 respectively in 5-fold cross validation. All of the above results demonstrated the superior prediction performance of SNMFMDA. We also conducted three different case studies on Esophageal Neoplasms, Breast Neoplasms and Lung Neoplasms, and 49, 49, and 48 of the top 50 of their predicted miRNAs respectively were confirmed by databases or related literatures. It could be expected that SNMFMDA would be a model with the ability to predict disease-related miRNAs efficiently and accurately.

## Introduction

MicroRNAs (miRNAs) are a class of endogenous non-coding RNAs with regulatory functions found in eukaryotes, which are approximately 20–25 nucleotides in length (Ambros, 2001). There are evidences manifesting that miRNAs are one of the most abundant gene regulatory molecules in multicellular organisms, which might affect the expression of many protein-coding genes and play an important regulatory role in animals and plants (Bartel, 2004). With more and more researchers being interested in miRNAs, the researches on miRNAs have been further deepened, and the number of discovered miRNAs is gradually increasing in recent years. The latest database records 24,521 microRNA loci in 206 species and 30,424 mature miRNAs after processing (Kozomara and Griffiths-Jones, 2014). Recently, it has been verified that miRNAs are crucial constituent in cells and may make an important impact in many important biological processes, including proliferation (Cheng et al., 2005), development (Karp and Ambros, 2005), differentiation (Miska, 2005), viral infection (Miska, 2005) and so on. So it is taken for granted that there are associations between miRNAs and the generation as well as development of a number of human diseases (Alvarez-Garcia and Miska, 2005). For instance, by targeting BCL6 corepressor like BCORL1, the migration and invasion of hepatocellular carcinoma (HCC) cells are restrained by mir-876-5p, which provides a new idea for the treatment of HCC (Xu et al., 2018). And it has been confirmed that miR-485-5p inhibits the development and improves the chemosensitivity of breast cancer by regulating survivin, which provides a potential method for addressing the chemoresistance of breast cancer (Wang et al., 2018). Therefore, it is of great significance to research the relations between diseases and miRNAs, which contributes to study the pathogenesis of the disease at the molecular level and makes a big difference in the early diagnosis of human diseases (Jiang et al., 2010). Using biological experiments to identify potential disease-related miRNAs is time-consuming and costly (Jiang et al., 2013), so it is imperative to use low-cost, high-efficiency methods to predict miRNAs associated with diseases. In recent years, since a large amount of biological data has been collected and organized into different databases, it is feasible and necessary to develop computational model to reveal potential disease-miRNA associations based on these databases (Chen, 2015).

In the last couple of years, more and more computational models for miRNA-disease associations prediction have been developed (Chen et al., 2017b). In the view of that miRNAs with similar functions tend to be involved with phenotypically similar diseases and vice versa, numerous computational methods have been proposed recently (Bandyopadhyay et al., 2010). Chen et al. (2016b) presented the model of Within and Between Score for MiRNA-Disease Association prediction (WBSMDA) to predict potential miRNAs associated with diseases. In this method, Within-Score and Between-Score about miRNAs and diseases were calculated by integrating miRNA functional similarity, disease semantic similarity, known miRNA-disease associations and Gaussian interaction profile kernel similarity, and then these two scores were combined to acquire the relation probability of miRNA-disease pair. Unfortunately, how to more reasonably integrate Within-scores and Between-score to calculate relevant probabilities remained unresolved. What's more, Chen et al. (Chen and Yan, 2014) also proposed Regularized Least Squares for MiRNA-Disease Association (RLSMDA) to reveal the unknown relations between miRNAs and diseases. RLSMDA was a semi-supervised method, so it didn't need negative samples. However, the optimal values of parameters in RLSMDA had not yet been obtained, which might affect the prediction performance. By implementing random walk on the miRNA–miRNA functional similarity network, Chen et al. (2012) developed another model named Random Walk with Restart for MiRNA–Disease Association (RWRMDA) to identify miRNAs related with diseases. The outstanding prediction performance of RWRMDA had been confirmed by a number of experiments, but there was still a main limitation in this model. RWRMDA couldn't be applied to predict the relations between miRNAs and new diseases without any known associated miRNAs. Later, Chen et al. (2017a) introduced a novel model called Ranking-based KNN for miRNA-Disease Association prediction (RKNNMDA) to uncover the potential associations between miRNAs and diseases by applying K-Nearest Neighbors (KNN) algorithm to obtain k-nearest-neighbors both for miRNAs and diseases. After resorting the k-nearest-neighbors based on the Support Vector Machine (SVM) ranking model, they got the ranking of association probability of disease-miRNA pairs by implementing weighted voting. RKNNMDA was capable of being implemented on new diseases, which overcame the biggest limitation of RWRMDA. It was a pity that there were also some limitations in this method. RKNNMDA could not be used to score all miRNAs based on the same criteria, especially for miRNAs with more known related diseases. Mørk et al. (2014) proposed a reliable calculation model of miRNA-Protein-Disease (miRPD) which didn't directly predict the miRNAs related with diseases but through proteins. The relations between miRNAs and diseases were uncovered by integrating the predicted and known miRNA–protein associations with the associations of protein–disease text mined from the literature. And the prediction performance of miRPD would be further improved if more involved datasets were taken into account. Xuan et al. (2015) developed a novel method of MIRNAs associated with Diseases Prediction (MIDP) to reveal the associations between miRNAs and diseases by implementing random walk on a miRNA functional similarity network where the similarity scores of miRNAs pairs were obtained through their related diseases. What's more, Xuan et al. also presented its extension method MIDPE to predict the miRNAs related with new diseases. In addition, by constructing a high-dimensional vector space to store the distribution information on miRNAs and diseases, Pasquier et al. (Pasquier and Gardes, 2016) proposed MiRAI to identify the miRNAs associated with diseases based on the similarity of the high-dimensional vectors composed of the distribution information on miRNAs and diseases. Xuan et al. (2013) presented an effective model named Human Disease-related MiRNA Prediction (HDMP) where the calculation method of miRNA functional similarity was improved by taking more related information into account. miRNAs in the same family or cluster were assigned higher weights since they were more likely to be associated with diseases with phenotype similarity. In this method, the sub-scores of the miRNA's *k* neighbors were equal to the product of the neighbor's weight and the miRNA functional similarity, and then by adding the sub-scores of *k* neighbors, the relevance score of a miRNA-disease pair was obtained. In addition, Chen et al. (2018a) proposed Network Distance Analysis for MiRNA-Disease Association prediction (NDAMDA) to detect the miRNAs associated with diseases. Compared to other methods, the improvement of NDAMDA lied in that in addition to the direct network distance between two studied diseases (miRNAs), the respective mean distances for each of them and all the rest of diseases (miRNAs) were taken into consideration. By implementing the matrix completion algorithm to update the adjacency matrix which recorded the known associations of disease-miRNA pairs in HMDD and then uncovering the unknown relations, Li et al. (2017) developed a method of Matrix Completion for MiRNA-Disease Association prediction model (MCMDA) without the need of negative samples. Compared with other computational models, the biggest advantage of MCMDA was that it only required known miRNA-disease associations, which also led to that MCMDA couldn't be introduced to predict potential associated miRNAs for new diseases or potential associated diseases for new miRNAs. Furthermore, the optimal parameters of this method were still unknown. In addition, by integrating miRNA functional similarity, disease semantic similarity, Gaussian interaction profile kernel similarity, and miRNA-disease associations confirmed by experiments into a heterogeneous graph, Chen et al. (2016c) presented a model of Heterogeneous Graph Inference for MiRNA-Disease Association prediction (HGIMDA) to reveal the unknown relations of miRNA-disease pairs by incorporating related data into a heterogeneous graph and summarizing all paths with the length equal to three to calculate the association probability of disease-miRNA pair. Unfortunately, limitations also existed in this method that for those miRNAs with more known related diseases, scores made by HGIMDA were generally higher than those miRNAs with less. Later, Chen et al. (2018b) proposed another method called Graph Regression for MiRNA-Disease Association prediction (GRMDA). In this method, by using two matrix decomposition methods to extract important correlation properties and filter noise, graph regression was performed synchronously in three potential spaces including the associated space, miRNA similarity space, and disease similarity space to reveal the potential disease-miRNAs associations. However, there were still some problems of GRMDA to be settled. For example, according to the size of the matrix, how to choose the optimal parameters in SVD and PLS remained unsolved. By optimizing the existing method for maximizing the flow of information, which was mainly used to prioritize disease-associated protein-coding genes, Yu et al. (2017) developed a combinatorial prioritization algorithm to predict the miRNA-disease associations. This method didn't require negative samples, which solved the problem that negative microRNA-disease associations were difficult to obtain.

In this paper, we proposed Symmetric Nonnegative Matrix Factorization for MiRNA-Disease Association prediction (SNMFMDA) to predict potential miRNA-disease associations. The process was mainly divided into two steps. Firstly, we used symmetric non-negative matrix factorization (SymNMF) to interpolate the integrated similarity matrix. Secondly, based on interpolated integrated similarity matrix, we utilized Kronecker regularized least square (KronRLS) method to obtained disease-miRNA association score matrix. We implemented global and local Leave-One-Out Cross Validation (LOOCV) and 5-fold cross validation to assess the prediction performance of SNMFMDA. As shown in the results, the AUC values of global LOOCV, local LOOCV, and 5-fold cross validation of SNMFMDA reached 0.9007, 0.8426, and 0.8830 ± 0.0017 respectively, which verified the excellent prediction performance of SNMFMDA.

## Materials and methods

### Human miRNA-disease association

In this paper, we obtained known human disease-miRNA associations from HMDD v2.0, which recorded 5430 experimentally verified associations between 383 diseases and 495 miRNAs. To better represent whether there were known associations between diseases and miRNAs, we defined a *nd* × *nm* adjacency matrix *A*, where *nd* and *nm* corresponded to the number of diseases and miRNAs respectively. If the relation between disease *d*(*i*) and miRNA *m*(*j*) had been verified, the value of the element *A*(*d*(*i*), *d*(*j*)) of the matrix was 1, otherwise 0.

### MiRNA functional similarity

On the basis of the assumption that functionally similar miRNAs tend to be associated with similar diseases and vice versa, miRNA functional similarities were calculated in this paper (Wang et al., 2010), and we could download them from http://www.cuilab.cn/fles/images/cuilab/misim.zip. For the sake of better describing the functional similarity between miRNAs, we defined the miRNA functional similarity matrix *FS*, where the element *FS*(*m*(*i*), *m*(*j*)) represented the functional similarity score between miRNA *m*(*i*) and *m*(*j*) (For the specific calculation process of miRNAs functional similarity, please see Supplementary Material).

### Disease semantic similarity model 1

To describe the association between diseases, the Directed Acyclic Graphs (DAGs) were built. *DAG*(*D*) = (*D, T*(*D*), *E*(*D*)) was applied to indicate disease *D*, where *T*(*D*) was a set of nodes composed of node *D* itself and its ancestor nodes and *E*(*D*) was a set consisting of edges directly from parent nodes to the child nodes (Wang et al., 2010). The contribution of disease *d* to the semantic value of disease *D* in DAG(*D*) and the semantic value of disease *D* were defined as follows:

(1) $$\left\{ \begin{array}{cc} D1_{D}(d)=1\; & ifd=D\\ D1_{D}(d)=\max\{\Delta *D1_{D}d|d\in children\;of\;d\} & if\;d\neq D \end{array}\right.$$

(2) $$\begin{array}{r} DV1\left(D\right)=\underset{d\in T\left(D\right)}{\sum }{D1}_{D}\left(d\right) \end{array}$$

where ▵ is the semantic contribution factor. The contribution of disease *D* to its own semantic value was 1, and the contribution of other diseases to the semantic value of disease *D* was negatively related to the distance between the disease and disease *D*, so the diseases in the same layer might have the same contribution to the semantic value of disease *D*.

Here, based on the model in the paper (Xuan et al., 2013), we constructed disease semantic similarity matrix *SS*1, whose element *SS*1(*d*(*i*), *d*(*j*)) indicated the semantic similarity score between disease *d*(*i*) and *d*(*j*). Based on the assumption that the more DAGs the two diseases overlapped, the greater their semantic similarity would be. The disease semantic similarity between disease *d*(*i*) and *d*(*j*) was calculated as follows:

(3) $$\begin{array}{c} SS1(d(i),d(j))=\frac{\sum_{t\in T(d(i))\cap^{\text{​}}T(d(j))}\left(D_{d(i)}(t)+D_{d(j)}(t)\right)}{DV1(d(i))+DV1(d(j))} \end{array}$$

### Disease semantic similarity model 2

For these diseases that appeared in the same layer of the DAG(*A*), according to the above definition of the disease semantic similarity model 1, they had the same contribution to the semantic value of the disease *A*. However, they might appear in different number of disease DAGs. For example, for two diseases *d*(*i*) and *d*(*j*) that appeared in the same layer of DAG(A), disease *d*(*i*) occurred in more disease DAGs, while *d*(*j*) appeared in less. It was clear that the contributions of the two diseases to the semantic value of disease *A* were different and the disease *d*(*i*) should have a less contribution to the semantic value of the disease *A* than *d*(*j*). Therefore, it was unreasonable to simply calculate the contribution to the semantic value of disease according to the definition of the disease semantic similarity model 1. Here, according to the model in the paper (Xuan et al., 2013), we defined the disease semantic similarity model 2 to supplement model 1. In the second model, diseases appeared in the same layer of DAG(*A*) might not necessarily ensure that they had the same contribution to the semantic value of disease *A*. The contribution of disease *D* to the semantic value of disease *A* was calculated as follows:

(4) $$\begin{array}{r} {D2}_{A}\left(D\right)=-\log\frac{the\;number\;of\;DAGs\;including\;D}{the\;number\;of\;diseases} \end{array}$$

The semantic value of disease *A* was calculated as follows:

(5) $$\begin{array}{r} DV2\left(A\right)=\sum_{t\in T\left(A\right)}{D2}_{A}\left(t\right) \end{array}$$

Similar to the disease semantic similarity matrix *SS*1, the element *SS*2(*d*(*i*), *d*(*j*)) of the disease semantic similarity matrix *SS*2 was calculated as follows:

(6) $$\begin{array}{c} SS2(d(i),d(j))=\frac{\sum_{t\in T(d(i))\cap^{\text{​}}T(d(j))}\left(D2_{d(i)}(t)+D2_{d(j)}(t)\right)}{DV2(d(i))+DV2(d(j))} \end{array}$$

Here, *SS*2(*d*(*i*), *d*(*j*)) was the disease semantic similarity between disease *d*(*i*) and *d*(*j*). Combining the two models of disease semantic similarity, we could calculate the final disease semantic similarity matrix *SS* as follow:

(7) $$\begin{array}{r} SS=\frac{SS1+SS2}{2} \end{array}$$

### Gaussian interaction profile kernel similarity

On the basis of the assumption that functionally similar miRNAs were more likely to be associated with similar diseases and vice versa, by taking the topological information of known miRNA-disease association network into account, we defined Gaussian interaction profile kernel similarity for diseases to describe the similarities between diseases based on the model in the paper (van Laarhoven et al., 2011). Here, we applied binary vector *IP*(*d*(*i*)) to represent the *ith* row of the adjacency matrix *A*, which recorded the association information of disease *d*(*i*) with all miRNAs. The Gaussian interaction profile kernel similarity matrix for diseases was defined as *KD*. The element *KD*(*d*(*i*), *d*(*j*)) indicated the Gaussian interaction profile kernel similarity between disease *d*(*i*) and *d*(*j*) and could be calculated as follows:

(8) $$\begin{array}{r} KD\left(d\left(i\right),d\left(j\right)\right)=\exp\left(-\gamma_{d}{\left||IP\left(d\left(i\right)\right)-\;IP\left(d\left(j\right)\right)|\right|}^{2}\right)\; \end{array}$$

Here, the role of parameter γ_*d*_ is to control the kernel bandwidth and it could be obtained by normalizing another new bandwidth parameter $\gamma_{d}^{{}^{\prime }}$ by the average number of associated miRNAs for all the diseases.

(9) $$\begin{array}{c} \gamma_{d}=\frac{\gamma_{d}^{{}^{\prime }}}{\frac{1}{nd}\sum_{nd}^{u=1}||IP\;\;(d(u))|{|}^{2}} \end{array}$$

Here, we set the value of $\gamma_{d}^{{}^{\prime }}$ to 1. Similarly, the Gaussian interaction profile kernel similarity matrix *KM* was defined as follows:

(10) $$\begin{array}{r} KM\left(m\left(i\right),m\left(j\right)\right)=\exp\left(-\gamma_{m}{\left||IP\left(m\left(i\right)\right)-\;IP\left(m\left(j\right)\right)|\right|}^{2}\right)\; \end{array}$$

The binary vector *IP*(*m*(*i*)) represents the *ith* column of the adjacency matrix *A*. Similar to γ_*d*_, parameters γ_*m*_ was calculated as follows:

(11) $$\gamma^{\prime }{}_{m}\frac{1}{nm}\overset{nm}{\underset{u=1}{\sum }}\|IP\;(m(u)){\|}^{2}$$

Here, the value of ${\gamma^{\prime }}_{m}$ was set to 1.

### Integrated similarity for miRNAs and diseases

As we know, many diseases can be described by a DAG. Based on the assumption that two diseases with large overlapping parts of their DAGs are considered to have large semantic similarity, we could calculate the semantic similarity between diseases, but we could not get DAG for all diseases, so for those diseases without DAG, we could not calculate the semantic similarity between them and other diseases. Therefore, we constructed integrated similarity matrix *SD* for diseases by integrating disease semantic similarity matrix and Gaussian interaction profile kernel similarity matrix in the following way according to the model in this paper (Chen et al., 2016b):

(12) $$\begin{array}{r} SD\left(d\left(i\right),d\left(j\right)\right)=\frac{SS\left(d\left(i\right),d\left(j\right)\right)+KD\left(d\left(i\right),d\left(j\right)\right)}{2} \end{array}$$

Similar to disease, the integrated similarity matrix *SM* was calculated as follow:

(13) $$\begin{array}{r} SM\left(m\left(i\right),m\left(j\right)\right)=\frac{FS\left(m\left(i\right),m\left(j\right)\right)+KM\left(m\left(i\right),m\left(j\right)\right)}{2} \end{array}$$

### SNMFMDA

**Figure 1:**
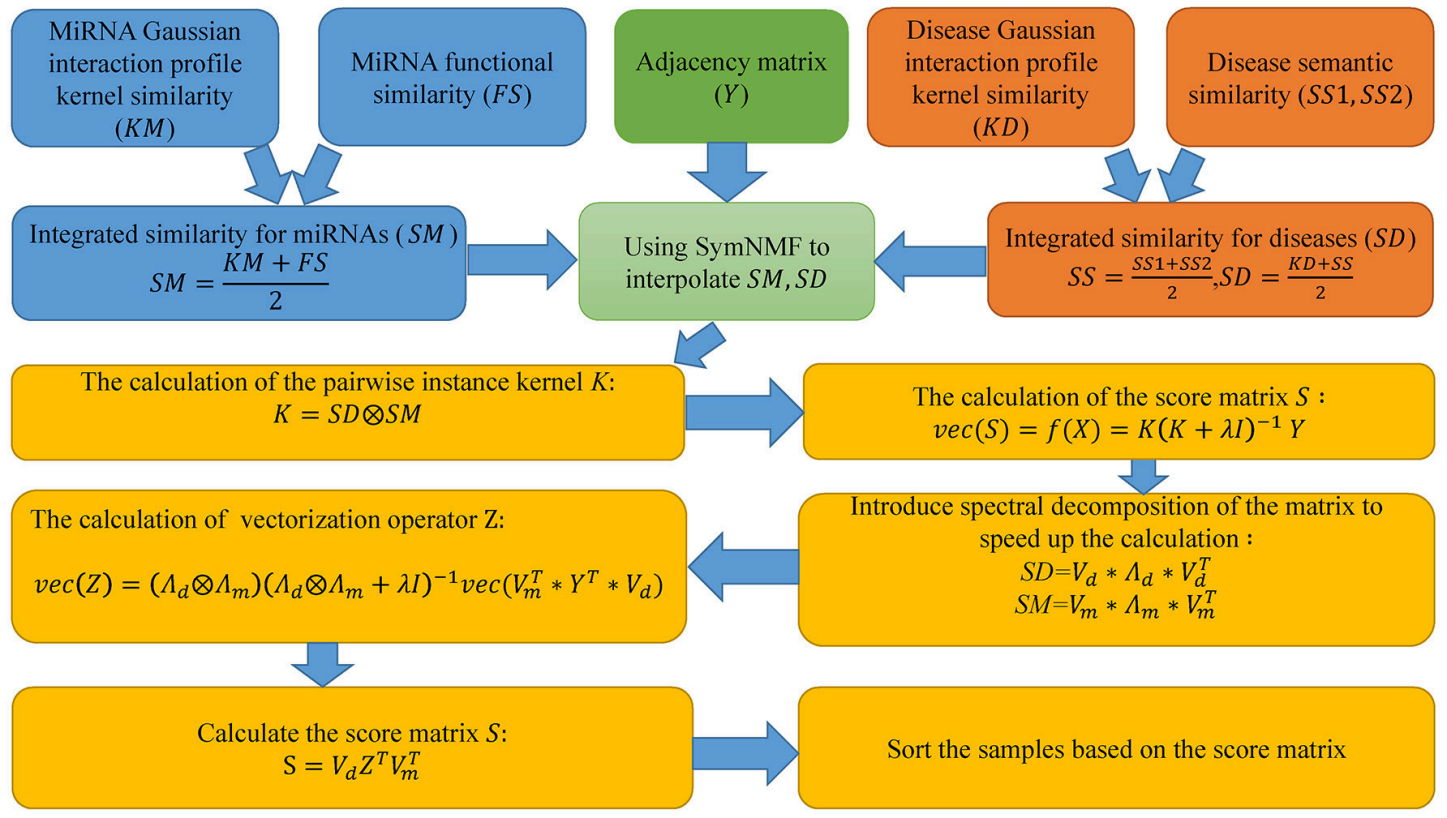
The flowchart of SNMFMDA included three steps: the integration of data; the calculation of the score matrix; the sorting of samples.

Motivated by the paper (Chen and Li, 2017), in this paper, we proposed SNMFMDA to predict potential miRNA-disease associations and the flow chart of the algorithm is shown in Figure 1. First step, we used SymNMF to interpolate the integrated similarity matrix *SM* and *SD*. Second step, based on interpolated integrated similarity matrix *SM, SD*, we utilized KronRLS method to obtained score matrix *S* with the same dimension as the adjacency matrix *A*, and each element of *S* was the associated probabilities of the corresponding disease-miRNA pairs. The two-step process was as follows:

### SymNMF

As an unsupervised learning method, non-negative matrix factorization (NMF) was extremely versatile and it had gradually become one of the most popular multidimensional data processing tools in signal processing, semantic analysis of documents and image engineering (He et al., 2011). In our model, we improved the integrated similarity by introducing SymNMF, which was a special kind of nonnegative matrix factorization. For the matrix *SD*, our purpose was to find a matrix *P* with the same size as the integrated similarity matrix *SD*, which also satisfied the following requirement:

(14) $$\begin{array}{r} SD\approx PP^{T} \end{array}$$

The specific process was as follows: The first step was initialization and we constructed a random matrix *P*^0^ whose elements were all positive as the initialization of the matrix *P*. *P*^*i*^ indicated the matrix corresponding to *P* in the beginning of the *ith* update, and the norm *E*^*i*^ in *ith* update could be computed as follow:

(15) $$\begin{array}{r} E^{i}=||SD-P^{i}{P^{i}}^{T}|{|}_{F}^{2} \end{array}$$

The second step was update. Here, we temporarily marked *P* as *P*^*new*^ after each update. The specific process was as follows:

(16) $$\begin{array}{rcl} R^{i} & = & \left(\left(SD\right)P^{i}\;\right)\;\cdot /\left(P^{i}{P^{i}}^{T}P^{i}\right) \end{array}$$

(17) $$\begin{array}{rcl} P^{new} & = & P^{i}.*\left(1-\alpha +\alpha {\cdot R}^{i}\right) \end{array}$$

(18) $$\begin{array}{rcl} {\;E}^{new} & = & ||SD-P^{new}{\left(P^{new}\right)}^{T}|{|}_{F\;}^{2\;} \end{array}$$

where *A*.^*^B and *A*./*B* were the entrywise product (i.e., Hadamard product) and entrywise division respectively. The value of α should be less than 1 but close to 1, here, we set α = 0.999. Then compared *E*^*new*^ to *E*^*i*^, if *EN* was smaller, update process ended, otherwise, updated *P* as follows:

(19) $$\begin{array}{rcl} P^{new} & = & P^{i}.^{*}{\left(R^{i}\right)}^{\frac{1}{3}} \end{array}$$

(20) $$\begin{array}{rcl} E^{new} & = & ||P^{new}{\left(P^{new}\right)}^{T}|{|}_{F}^{2} \end{array}$$

(21) $$\begin{array}{rcl} P^{new} & \to & P^{i+1} \end{array}$$

(22) $$\begin{array}{rcl} E^{new} & \to & E^{i+1} \end{array}$$

(23) $$\begin{array}{rcl} i & = & i+1 \end{array}$$

After that, the procedure went back to formula (15) and started the next update. Using SymNMF to perform interpolation on the similarity matrix *SM* was similar to *SD*.

### KronRLS

First of all, we needed to build a list *D* = [*d*(1), *d*(2), …*d*(*nd*)] for diseases. Similarly, list *M* = [*m*(1), *m*(2), …*m*(*nm*)] was constructed to denoted *nm* miRNAs. All the columns of adjacency matrix *A* were stitched together to form a *n*-dimensional column vector *Y* where *n* = *nd*×*nm* indicated the total number of disease-miRNA pairs. And then we built another *n*-dimensional column vectors *X* whose element *X*_*i*_ represented the disease-miRNA pair corresponding to the *ith* element in *Y*. The purpose of the RLS algorithm was to find the mapping function *f* from vector *X* to score matrix *S* by minimizing the following function.

(24) $$\begin{array}{r} J\left(f\right)=\frac{1}{2n}\overset{n}{\underset{i=1}{\sum }}{\left(Y_{i}-f\left(X_{i}\right)\right)}^{2}+\frac{\lambda }{2}||f|{|}_{K}^{2} \end{array}$$

where ||*f*||_*K*_ is a norm of function *f* on the Hilbert space related with the kernel *K*. λ is a regularization parameter that determine the trade-off between prediction error and model complexity, here, we set it to 1. The representative theorem ensured that the Equation (23) had a closed form solution:

(25) $$\begin{array}{r} f\left(X\right)=\overset{n}{\underset{i=1}{\sum }}a_{i}K\left(X,X_{i}\right)=Ka \end{array}$$

Here, *a* is also an *n*-dimensional vector and it could be get by solving the following equation:

(26) $$\begin{array}{r} \left(K+\lambda I\right)a=Y \end{array}$$

where *I* is the identity matrix and *K* is named as the pairwise instance kernel to represent the similarity of two data points in the Hilbert space. To be specific, for two disease-miRNA pairs (*d*_*i*_, *m*_*j*_) and (*d*_*w*_, *m*_*z*_), *K*((*d*_*i*_, *m*_*j*_), (*d*_*w*_, *m*_*z*_)) indicates the similarity between the two disease-miRNA pairs. And the kernel could be calculated as follow:

(27) $$\begin{array}{rcl} K\left(\left(d_{i},m_{j}\right),\left(d_{w},m_{z}\right)\right) & = & SD\left(i,w\right)SM\left(j,z\right) \end{array}$$

(28) $$\begin{array}{rcl} K & = & SD⊗SM \end{array}$$

where *SD* ⊗ *SM* is the Kronecker product of *SD* and *SM*, and the relation probabilities of all disease-miRNA pairs could be obtained according to the kernel as follow:

(29) $$\begin{array}{r} vec\left(S\right)=f\left(X\right)=K{\left(K+\lambda I\right)}^{-1}\;Y \end{array}$$

Here, *vec*(·) is a vectorization operator that combine all the columns of a matrix into a column vector.

In order to solve the problem more effectively, we introduced spectral decomposition of the matrix to speed up the calculation. The decompositions of integrated similarity *SD, SM and K* were defined as follow:

(30) $$\begin{array}{rcl} SD & = & \vee_{d}\wedge_{d}\vee_{d}^{T} \end{array}$$

(31) $$\begin{array}{rcl} SM & = & \vee_{m}\wedge_{m}\vee_{m}^{T} \end{array}$$

(32) $$\begin{array}{rcl} K & = & \vee \wedge \vee^{T} \end{array}$$

Here, the dimension of the matrix ∨_*d*_(∨_*m*_, ∨) is the same as *SD*(*SM, K*), and each of its columns is the eigenvector of the matrix *SD*(*SM, K*). Matrix ∧_*d*_(∧_*m*_, ∧) is a diagonal matrix whose diagonal element ∧_*d*_*ii*__(∧_*m*_*ii*__, ∧_*ii*_) is the eigenvalue of *SD*(*SM, K*) corresponding to the *ith* column [i.e., the *ith* eigenvector of *SD*(*SM, K*)]. Then, the Kronecker product of *SD* and *SM* could be calculated as follows:

(33) $$\begin{array}{rcl} K=SD⊗SM & = & \vee \wedge \vee^{T} \end{array}$$

(34) $$\begin{array}{rcl} K{\left(K+\lambda I\right)}^{-1} & = & \vee \wedge {\left(\wedge +\lambda I\right)}^{-1}\vee^{T} \end{array}$$

where:

(35) $$\begin{array}{rcl} \vee & = & \vee_{d}⊗\vee_{m} \end{array}$$

(36) $$\begin{array}{rcl} \wedge & = & \wedge_{d}⊗\wedge_{m} \end{array}$$

Here, we introduced a property of the Kronecker product:

(37) $$\begin{array}{r} \left(A⊗B\right)vec\left(Y\right)=vec\left(BYA^{T}\right) \end{array}$$

By integrating the above formulas, the score matrix *S* could be calculated as follows:

(38) $$\begin{array}{r} S=V_{d}Z^{T}V_{m}^{T} \end{array}$$

where:

(39) $$\begin{array}{r} vec\left(Z\right)=\left(\Lambda_{d}⊗\Lambda_{m}\right){\left(\Lambda_{d}⊗\Lambda_{m}+\lambda I\right)}^{-1}vec\left(V_{m}^{T}Y^{T}V_{d}\right) \end{array}$$

## Results

### Performance evaluation

In this study, based on the 5,430 confirmed associations between 383 diseases and 495 miRNAs recorded in HMDD v2.0, local and global LOOCV were applied to test the prediction performance of SNMFMDA. In LOOCV, each of the 5,430 known associations (positive samples) was left out in turn as the test sample and the remaining 5,429 known associations were considered as training samples, while the miRNA-disease pairs without verified association were viewed as candidate samples (unknown samples), and then we applied SNMFMDA to calculated the association probability of candidate samples and test sample. In global LOOCV, the score of test sample was ranked with all of the candidate samples, while we sort it with all the unknown samples that contained the studied diseases in local LOOCV. When the ranking of test sample was higher than the given threshold, we affirmed that SNMFMDA had correctly predicted the sample. We set the true positive rate (TPR, sensitivity) as the vertical axis and the false positive rate (FPR, 1-specificity) as the horizontal axis. When the thresholds took different values, they correspond to different points in the coordinate system. The bight composed of all these points was the Receiver operating characteristics (ROC) curve. Here, Sensitivity was the ratio of the number of correctly predicted test samples to the total number of positive samples, and specificity was the percentage of candidate samples whose ranking were lower than the given threshold to all of the unknown samples. The area under the ROC curve (AUC) was calculated to evaluate the reliability of SNMFMDA. The AUC value of 0.5 meant that the computational model was equivalent to random prediction, and AUC = 1 indicated that the prediction performance of the calculation model was excellent. In other words, when the value of AUC was greater than 0.5 and less than 1, the larger the value, the better the prediction performance.

**Figure 2:**
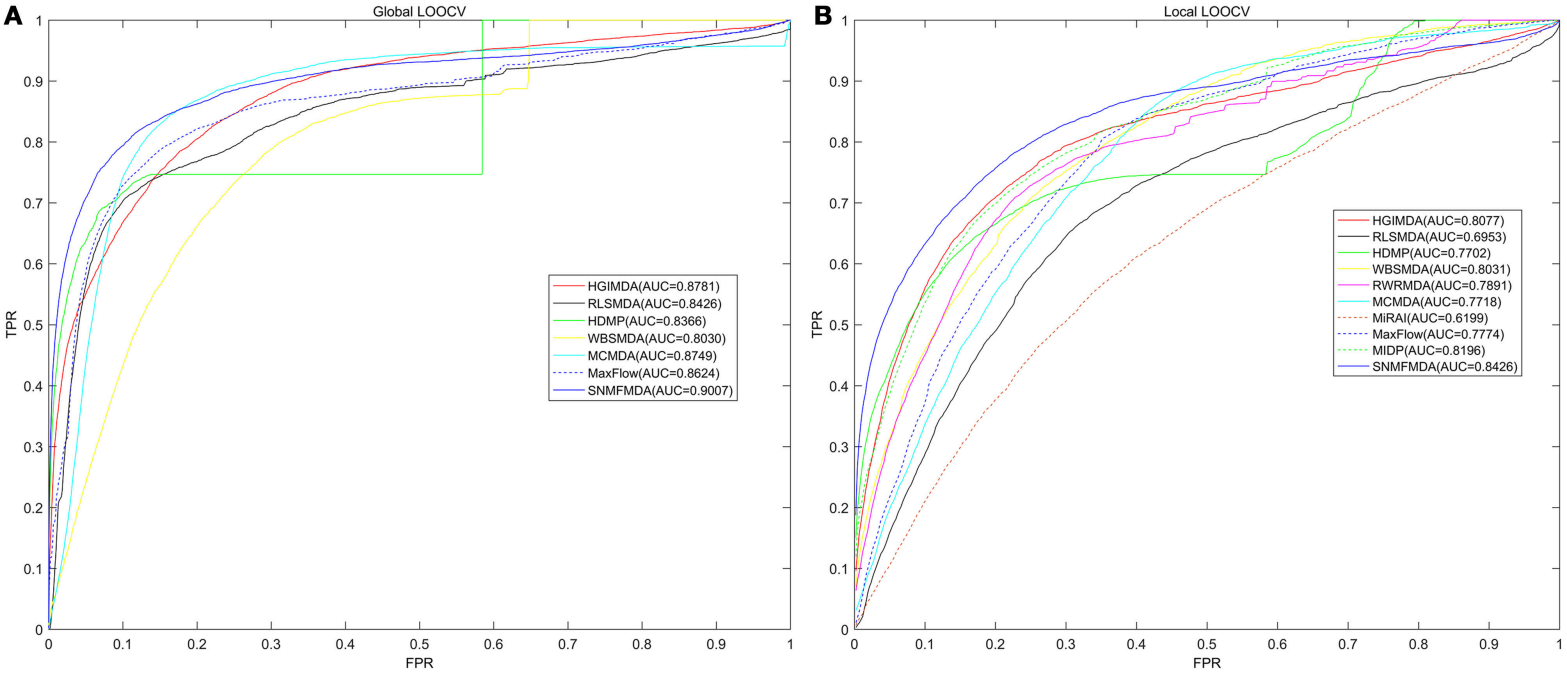
Comparison of prediction performance between SNMFMDA and other computation models (HGIMDA, RLSMDA, HDMP, WBSMDA, MCMDA, MaxFlow, MiRAI, MIDP) based on global **(A)** and local **(B)** cross validation results. As shown in the figure, the AUC value of global and local LOOCV of SNMFMDA reached 0.9007 and 0.8426, which fully proved the superior prediction performance of SNMFMDA.

The comparison of the prediction performance between a couple of computational methods based on the AUC value of global and local LOOCV respectively was shown in Figure 2. As a consequence, the AUC of SNMFMDA was 0.9007, while the AUC values of HGIMDA (Chen et al., 2016c), MCMDA (Li et al., 2017), MaxFlow (Yu et al., 2017), RLSMDA (Chen and Yan, 2014), HDMP (Xuan et al., 2013), WBSMDA (Chen et al., 2016b) were respectively 0.8781, 0.8749, 0.8624, 0.8426, 0.8366, 0.8030 in global LOOCV. In local LOOCV, SNMFMDA obtained AUC of 0.8426, which were clearly better than HGIMDA (0.8077), MCMDA (0.7718), MaxFlow (0.7774), RLSMDA (0.6953), HDMP (0.7702), WBSMDA (0.8031), MiRAI (0.6299), MIDP (0.8196), and RWRMDA (0.7891). Both RWRMDA and MIDP weren't capable to predict potential related miRNAs for all diseases at the same time, so we could only evaluate their prediction performance with local LOOCV instead of global LOOCV. Besides, the association probabilities of candidate samples calculated by MiRAI had a high-positive correlation with the number of known associations of corresponding diseases. The more known miRNAs associated with a disease, the greater the disease-related candidate samples' association probabilities would be. Thus, it wasn't reasonable to compare the association probabilities of candidate samples corresponding to different diseases. Therefore, we couldn't apply global LOOCV to evaluate the prediction performance of RWRMDA, MIDP, and MiRAI. What's more, as could be seen from Figure 2, the value of the AUC for local LOOCV of MiRAI was relatively small. This was because that the core of MiRAI was collaborative filtering which caused its prediction accuracy to heavily depend on the number of known miRNA-disease associations. The database used in our method had 383 diseases but there were few known miRNAs associated with each disease. Therefore, the predictive performance of MiRAI based on this database was far worse than that in the original literature where the training database contained more verified associations for each disease.

In addition, we also applied 5-fold cross-validation to evaluate the predictive performance of SNMFMDA. All known miRNA-disease associations were randomly divided into five equal parts, and each part was in turn treated as the test sample while the other four parts were treated as the training samples. Besides, in order to reduce the influence of the division of known associations on prediction accuracy, we performed 100 random divisions. It could be seen from the results that the mean and standard deviation of the AUC in 5-fold cross validation respectively reached 0.8830 and 0.0017, which was obviously better than MCMDA (0.8767 ± 0.0011), MaxFlow (0.8579 ± 0.001), RLSMDA (0.8569 ± 0.0020), HDMP (0.8342 ± 0.0010), and WBSMDA (0.8185 ± 0.0009). In summary, all cross-validation results further proved the superior prediction performance of SNMFMDA.

### Case studies

In addition to cross-validation, we also used case studies to evaluate the prediction accuracy of our computational model. Here, we applied three different case studies on Esophageal Neoplasms (EN), Breast Neoplasms (BN), and Lung Neoplasms (LN) to test the prediction performance from different aspects. In the first kind, we could obtain 5,430 known miRNA-disease associations (positive samples) from HMDD v2.0 (Li et al., 2014) for 383 diseases and 495 miRNAs, and the remaining 184,155 samples were unknown samples. In order to predict the potential associations of miRNA-disease pairs from unknown samples, we scored them by SNMFMDA and ranked them. Finally, we verified the top 50 potential associated miRNAs for the investigated disease by two other databases dbDEMC (Yang et al., 2010) and miR2Disease (Jiang et al., 2009) that recorded a number of verified miRNA-disease associations. In order to assess SNMFMDA's prediction power for new diseases without any known associated miRNAs, the second kind of case study was implemented. Here, when we studied a disease, we first removed all its known associations based on the HMDD v2.0 that was, all the 1 s in the row corresponding to this disease in the adjacency matrix were turned to 0. After that we used SNMFMDA to score all miRNAs for the investigated disease and then sorted these miRNAs. Finally, we verified whether the associations between the disease and the top 50 miRNAs were verified by the three databases dbDEMC, miR2Disease and HMDD v2.0. Because there are a number of databases in which known miRNA-diseases associations were recorded, when the prediction accuracy of computation method based on a certain database was good, it couldn't explain that the prediction performance of the method was superior. To prove the applicability of our model to different databases, we carried out the third type of case studies. The third case study was similar to the first, and the difference between them was that the dataset on which SNMFMDA was based was not the same. There were 1895 verified associations between 137 diseases and 271 miRNAs recorded in the database HMDD v1.0 used in the third case study. Finally, we applied three databases (HMDD v2.0, miR2Disease and dbDEMC) to verify the top 50 predicted miRNAs for the investigated disease after utilizing our calculation model.

As one of the most common tumors in the world, Esophageal Neoplasms (EN) has the top 10 morbidity and mortality in all cancers (He et al., 2012). According to the latest estimates of related departments in US, in 2018, there would be 12,850 patients dying from EN, accounting for 4% of all patients dying of cancer (Siegel et al., 2018). Although the treatment method have been improved, the damage of EN to human have not been significantly reduced (He et al., 2012). The survival rate of patients with EN was less than 25% in the last five years (Kim et al., 2011). Research showed that if EN could be diagnosed early, its mortality rate was expected to drop to 10% (Daly et al., 2000), so finding better and more efficient diagnosis and treatment was imperative (Xie et al., 2013). More and more researches indicated that there was a close relationship between miRNAs and the development of human diseases (Alvarez-Garcia and Miska, 2005). A number of associations between miRNAs and EN had been verified. For instance, by sponging miR-200a, which was functionally similar to a competitive endogenous RNA, lncRNA MALAT1 adjusted the expression of ZEB1 and ZEB2 to facilitate the invasion and migration of EN cells by mean of inducing epithelial-mesenchymal transition (Zhang et al., 2017). We used SNMFMDA to perform the first case study on EN, and the results showed that 47 of the top 50 predicted EN-related miRNAs were verified by other two databases dbDEMC and miR2Disease. For the remaining three miRNAs that had not been verified by the above two databases, there were studies showing that serum expression levels of mir-218 (15th in the prediction list) in EN patients were significantly lower than those in healthy people, and the levels were related to tumor differentiation, staging, and lymph node metastasis. For this reason, mir-218 was highly likely to be the target of the early diagnosis of EN, which provided a new idea for the detection of this cancer (Jiang et al., 2015). In EN cells, mir-122 (43th in the prediction list) targeted pyruvate kinase M2 (PKM2), and Tanshinone IIA could limit the expression of PKM2 by promoting the expression of mir-122, which in turn restricted the growth of EN cells (Zhang et al., 2016). As could be seen from the verification results, only one of the top 50 predicted miRNAs had not been validated by the database or literature (see Table 1).

**Table 1:** Prediction of the top 50 predicted miRNAs associated with Esophageal Neoplasms.

**miRNA**	**Evidence**	**miRNA**	**Evidence**
hsa-mir-18a	dbDEMC	hsa-mir-29a	dbDEMC
hsa-mir-200b	dbDEMC	hsa-mir-106a	dbDEMC
hsa-mir-1	dbDEMC	hsa-mir-10b	dbDEMC
hsa-mir-17	dbDEMC	hsa-mir-191	dbDEMC
hsa-mir-19b	dbDEMC	hsa-mir-497	dbDEMC
hsa-mir-125b	dbDEMC	hsa-mir-9	dbDEMC
hsa-let-7d	dbDEMC	hsa-let-7f	Unconfirmed
hsa-mir-142	dbDEMC	hsa-mir-132	dbDEMC
hsa-let-7e	dbDEMC	hsa-mir-424	dbDEMC
hsa-mir-16	dbDEMC	hsa-mir-146b	dbDEMC
hsa-mir-199b	dbDEMC	hsa-mir-224	dbDEMC
hsa-mir-125a	dbDEMC	hsa-mir-151	dbDEMC
hsa-mir-194	dbDEMC;miR2Disease	hsa-mir-24	dbDEMC
hsa-mir-429	dbDEMC	hsa-mir-182	dbDEMC
hsa-mir-218	PMID: 25812647	hsa-mir-106b	dbDEMC
hsa-mir-221	dbDEMC	hsa-mir-181b	dbDEMC
hsa-let-7i	dbDEMC	hsa-mir-7	dbDEMC
hsa-mir-195	dbDEMC	hsa-mir-122	PMID: 27040384
hsa-mir-30a	dbDEMC	hsa-mir-335	dbDEMC
hsa-mir-222	dbDEMC	hsa-mir-302c	dbDEMC
hsa-mir-107	dbDEMC;miR2Disease	hsa-mir-302b	dbDEMC
hsa-mir-30c	dbDEMC	hsa-let-7g	dbDEMC
hsa-mir-18b	dbDEMC	hsa-mir-181a	dbDEMC
hsa-mir-133b	dbDEMC	hsa-mir-491	dbDEMC
hsa-mir-127	dbDEMC	hsa-mir-32	dbDEMC

*The first 25 miRNAs and the last 25 miRNAs were recorded in the first and third columns, respectively. The second and forth columns recorded the database or literatures in PubMed that verified the corresponding miRNAs associated with Esophageal Neoplasms*.

In order to facilitate further validation and research, we have provided the complete prediction list of potential miRNAs associated with all the 383 human diseases in HMDD v2.0 (see Supplementary Table 1).

Breast Neoplasms (BN) is a type of cancer with high morbidity and mortality among women in the United States (Kelsey and Horn-Ross, 1993). According to the prediction of relevant departments, 40,920 women would die from BN in the United States in 2018, accounting for 14% of the total cancer deaths (Siegel et al., 2018). According to the current medical level, the only way to improve the cure rate and reduce the mortality rate of BN lies in the early detection and timely treatment (Tao et al., 2015). In order to improve the diagnostic efficiency, the researchers have put forward many methods including the prediction of potential relevant miRNAs of BN. It has been proved that the expression levels of mir-21 and mir-146a in plasma samples of BN patients were obviously higher than that of healthy volunteers and we could identify whether a patient had BN based on the expression level of mir-21 and mir-146a in plasma (Kumar et al., 2013). We implemented the second case study on BN and 45 of the top 50 miRNAs potentially associated with BN were verified by databases (HMDD, dbDEMC and miR2Disease). Among the remaining 5 miRNAs that were not validated by the databases, the expression of mir-142 (12th in the prediction list) was dysregulated in BN cells and mir-142 could restricted the invasion of BN cells by simultaneously targeting WASL, Integrin Alpha V, and Additional Cytoskeletal Elements (Schwickert et al., 2015). The expression of mir-378a-3p (20th in the prediction list) was lower in BN tissues, and it acted on the endocrine resistance mechanism of BN by regulating the expression of GOLT1A (Ikeda et al., 2015). It was also verified that there was excessive expression of mir-302f (48th in the prediction list) in HER2-postive BN (Kang et al., 2014). What's more, researches also confirmed that there was overexpression of mir-744 (49th in the prediction list) in BN cell and mir-744 played a part in the drug resistance of BN (Chen et al., 2016a). The above results (see Table 2) showed that 49 of the top 50 potential BN-associated miRNAs predicted by SNMFMDA were validated, which indicated that the prediction performance of SNMFMDA was still reliable when it was applied to new diseases.

**Table 2:** Prediction of the top 50 predicted miRNAs associated with Breast Neoplasms.

**miRNA**	**Evidence**	**miRNA**	**Evidence**
hsa-mir-21	dbDEMC;miR2Diseaes;HMDD	hsa-mir-200c	dbDEMC;miR2Diseaes;HMDD
hsa-mir-125b	miR2Disease;HMDD	hsa-mir-221	dbDEMC;miR2Diseaes;HMDD
hsa-mir-31	dbDEMC;miR2Diseaes;HMDD	hsa-mir-708	HMDD
hsa-mir-99a	dbDEMC	hsa-mir-218	dbDEMC;HMDD
hsa-mir-375	dbDEMC;HMDD	hsa-mir-205	dbDEMC;miR2Diseaes;HMDD
hsa-mir-146a	dbDEMC;miR2Diseaes;HMDD	hsa-mir-629	dbDEMC;HMDD
hsa-mir-100	dbDEMC;HMDD	hsa-mir-101	dbDEMC;miR2Diseaes;HMDD
hsa-mir-302b	dbDEMC;HMDD	hsa-mir-193b	dbDEMC;miR2Diseaes;HMDD
hsa-mir-302c	dbDEMC;HMDD	hsa-mir-197	dbDEMC;HMDD
hsa-let-7a	dbDEMC;miR2Diseaes;HMDD	hsa-mir-370	dbDEMC
hsa-mir-138	dbDEMC	hsa-mir-148a	dbDEMC;miR2Diseaes;HMDD
hsa-mir-142	PMID: 26657485	hsa-mir-27a	dbDEMC;miR2Diseaes;HMDD
hsa-mir-486	dbDEMC;HMDD	hsa-mir-34c	dbDEMC;HMDD
hsa-mir-7	dbDEMC;miR2Diseaes;HMDD	hsa-mir-196a	dbDEMC;miR2Diseaes;HMDD
hsa-mir-203	dbDEMC;miR2Diseaes;HMDD	hsa-mir-503	dbDEMC
hsa-mir-302d	dbDEMC;HMDD	hsa-mir-34b	dbDEMC;HMDD
hsa-mir-27b	dbDEMC;HMDD	hsa-let-7g	dbDEMC;HMDD
hsa-mir-302a	dbDEMC;HMDD	hsa-mir-151a	HMDD
hsa-mir-133b	dbDEMC;HMDD	hsa-mir-34a	dbDEMC;HMDD
hsa-mir-378a	PMID: 26255816	hsa-mir-642a	Unfirmed
hsa-mir-145	dbDEMC;miR2Diseaes;HMDD	hsa-mir-663a	HMDD
hsa-mir-9	dbDEMC;miR2Diseaes;HMDD	hsa-mir-151b	HMDD
hsa-mir-499a	HMDD	hsa-mir-302f	PMID: 24982406
hsa-let-7b	dbDEMC;HMDD	hsa-mir-744	PMID: 27746365
hsa-mir-574	dbDEMC	hsa-mir-451a	HMDD

*Here, all known associations with Breast Neoplasms had been removed. The first 25 miRNAs and the last 25 miRNAs were recorded in the first and third columns, respectively. The second and forth columns recorded the database or literatures in PubMed that verified the corresponding miRNAs associated with Breast Neoplasms*.

Lung Neoplasms (LN) is one of the most deadly cancers (Liu and Wei, 2018), and its poor prognosis have also caused great harm to human health (Zhao et al., 2018). According to the estimation of the American Cancer Society, in 2018, new cases of bronchus and LN would reach 121,680, accounting for 14% of all newly diagnosed cancer patients, and it was estimated that there would be 83550 patients dying from lung cancer, more than a quarter of the total deaths (Siegel et al., 2018). The early diagnosis and treatment of LN is very difficult, fortunately, more and more studies have shown that miRNAs are closely related to the development, progression, and progression of LN (Zhao et al., 2018). For example, researches showed that there was significantly higher expression of mir-221 in LN patients than that in healthy people, and biological analysis indicated that the target of mir-221 was most likely related to the formation and development of LN (Zhu et al., 2017). Besides, mir-221 was very likely to become a non-aggressive biomarker for the diagnosis of LN (Zhu et al., 2017). As an embryo-expressing lung miRNA, mir-127 had been shown to be closely linked to the poor prognosis of LN (Shi et al., 2017). Therefore, the prediction of miRNAs associated with LN could enable us to understand the pathogenesis of cancer and might provide novel diagnostic methods and treatment approaches. We applied SNMFMDA to perform the third case study on LN to test the prediction power of the model when it is applied to another database HMDD v1.0. The prediction result showed that 44 of the top 50 potential LN-associated miRNAs were verified by other databases (dbDEMC, miR2Diseaes, HMDD v2.0). For the remaining 6 miRNAs that weren't verified by the three databases, studies showed that the mir-92 (6th in the prediction list) family was less expressed in cisplatin-resistant cells, which indicated that the mir-92 family played a part in the regulation of cisplatin resistance in non-small cell lung cancer (Zhao et al., 2015). Some researches confirmed that the overexpression of mir-194 (20th in the prediction list) produced an effect on the expression of Mpl/ERK pathway proteins and restrained the mitosis and proliferation of non-small cell lung cancer cells by targeting Human nuclear distribution C (hNUDC), which provided a novel strategy for the treatment of LN (Zhou et al., 2016). Studies confirmed that mir-372-3p (38th in the prediction list) was obviously overexpressed in lung squamous cell carcinoma cells and limited the expression of FGF9 by binding to it, which contributed to the proliferation of lung squamous cell carcinoma cells (LSCC). In contrast, the low expression of mir-372-3p or high expression of FGF9 were conducive to inhibit the growth and invasion of LSCC cells (Wang et al., 2017). The expression level of mir-320 (46th in the prediction list) in non-small cell lung cancer (NSCLC) cells was lower than the level in normal cells, and mir-320 limited cell growth in NSCLC cells through targeting fatty acid synthase (Lei et al., 2016). Based on the above results (see Table 3), 48 of the top 50 potential LN-associated miRNAs predicted by SNMFMDA were validated, which indicated that the prediction performance of the model based on other datasets was also very reliable.

**Table 3:** Prediction of the top 50 predicted miRNAs associated with Lung Neoplasms based on known associations in HMDD v1.0.

**miRNA**	**Evidence**	**miRNA**	**Evidence**
hsa-mir-221	dbDEMC;HMDD	hsa-mir-7	miR2Disease;HMDD
hsa-mir-127	dbDEMC;HMDD	hsa-mir-451	dbDEMC;miR2Disease
hsa-mir-200b	dbDEMC;miR2Diseaes;HMDD	hsa-mir-99b	Unfirmed
hsa-mir-16	dbDEMC;miR2Disease	hsa-mir-93	dbDEMC;miR2Diseaes;HMDD
hsa-mir-222	dbDEMC;HMDD	hsa-mir-18b	HMDD
hsa-mir-92b	PMID: 26482648	hsa-mir-196b	dbDEMC
hsa-mir-195	dbDEMC;miR2Disease	hsa-mir-100	dbDEMC;HMDD
hsa-mir-106b	dbDEMC	hsa-mir-200a	dbDEMC;miR2Diseaes;HMDD
hsa-mir-181b	dbDEMC;HMDD	hsa-mir-429	dbDEMC;miR2Disease
hsa-mir-141	dbDEMC;miR2Disease	hsa-mir-98	dbDEMC;miR2Diseaes;HMDD
hsa-mir-107	dbDEMC;HMDD	hsa-mir-23b	dbDEMC
hsa-mir-25	dbDEMC;HMDD	hsa-mir-10b	dbDEMC;HMDD
hsa-mir-15a	dbDEMC	hsa-mir-372	PMID: 28440022
hsa-mir-20b	dbDEMC	hsa-mir-135a	dbDEMC;HMDD
hsa-mir-148a	dbDEMC;HMDD	hsa-mir-186	dbDEMC;HMDD
hsa-mir-15b	dbDEMC	hsa-mir-181a	dbDEMC;HMDD
hsa-mir-133a	dbDEMC;HMDD	hsa-mir-22	miR2Disease;HMDD
hsa-mir-152	dbDEMC	hsa-mir-31	dbDEMC;miR2Diseaes;HMDD
hsa-mir-148b	dbDEMC	hsa-mir-339	dbDEMC;miR2Disease
hsa-mir-194	PMID: 27035759	hsa-mir-498	dbDEMC
hsa-mir-200c	dbDEMC;miR2Diseaes;HMDD	hsa-mir-320	PMID: 27277534
hsa-mir-206	HMDD	hsa-mir-181d	dbDEMC
hsa-mir-135b	dbDEMC;HMDD	hsa-mir-130b	dbDEMC
hsa-mir-296	dbDEMC	hsa-mir-103	Unfirmed
hsa-mir-373	dbDEMC	hsa-mir-302c	dbDEMC

*The first 25 miRNAs and the last 25 miRNAs were recorded in the first and third columns, respectively. The second and forth columns recorded the database or literatures in PubMed that verified the corresponding miRNAs associated with Lung Neoplasms*.

## Discussion

As accumulating studies have demonstrated that miRNAs play an extremely important role in human physiological processes, researches on the association between miRNAs and diseases have attracted more and more attention. Since it is time-consuming and costly to use biological experiments to reveal potential miRNA-disease associations, many computational models have been proposed to predict disease-related miRNAs in recent years. In the paper, we developed a novel model of SNMFMDA to reveal the relation of miRNA-disease pairs by integrating the known miRNA-disease associations recorded in HMDD v2.0, miRNA functional similarity, disease semantic similarity, and Gaussian interaction profile kernel similarity for diseases and miRNAs. SNMFMDA overcame the limitation of many previous models that they were incapable of predicting miRNAs associated with new diseases. As shown in the prediction results, the AUC values of global LOOCV, local LOOCV, and 5-fold cross validation reached 0.9007, 0.8426, and 0.8830 ± 0.0017 respectively. As we all know, models with global AUC value above 0.9 were rare, so SNMFMDA is a model with higher credibility. In the future, our model would be an effective tool to reveal potential disease-related miRNAs, which is conducive to the diagnosis and treatment of diseases.

The superior prediction performance of the model was mainly due to the following aspects. Firstly, the database on which SNMFMDA was based was reliable and the model could be used to predict miRNAs potentially associated with new diseases by introducing the information of disease similarity. Secondly, we used SymNMF to interpolate the integrated similarity, while many of the previous methods used the integrated similarity directly. Finally, we introduced spectral decomposition to speed up the calculation of the Kronecker product in our model. Certainly, there are still some limitations to be resolved in the future. For example, SNMFMDA might not score miRNAs using the same criteria, especially for those with more known related diseases. Although the prediction accuracy of SNMFMDA is obviously higher than many previous calculation methods, if the biological database on which our model is based can be further improved, SNMFMDA's prediction performance would be better. The calculation of disease similarity and miRNA similarity used in our model may not be the most perfect method, and we expect to add more biological data sets in future calculations to improve the accuracy of similarity calculations. Besides, SNMFMDA involved calculating the Kronecker product of two matrices. The solution of the Kronecker product of two matrices was equivalent to that each element of the previous matrix multiplied by the next matrix, so the sizes of the Kronecker product was much larger than the first two matrices. Therefore, calculating the Kronecker product often led to memory problems in computer. In addition, our model SNMFMDA did not consider miRNA-protein association and miRNA-cellular pathway association, which significantly affected the prediction performance of the model.

## Author contributions

YZ implemented the experiments, analyzed the result, and wrote the paper. XC conceived the project, developed the prediction method, designed the experiments, and revised the paper. JY analyzed the result and revised the paper. All authors read and approved the final manuscript.

### Conflict of interest statement

The authors declare that the research was conducted in the absence of any commercial or financial relationships that could be construed as a potential conflict of interest.
