# Supplementary material for: A Novel Computational Method for the Identification of Potential miRNA-Disease Association Based on Symmetric Non-negative Matrix Factorization and Kronecker Regularized Least Square
Source: Front Genet. 2018 Aug 21;9:324. doi: 10.3389/fgene.2018.00324 (PMC6111239; doi:10.3389/fgene.2018.00324)
Supplement: Supplementary file 2 [file Data_Sheet_1.docx]

**A novel computational method for the identification of potential miRNA-disease association based on symmetric non-negative matrix factorization and Kronecker regularized least square**

**Yan Zhao^1^, Xing Chen^1,*^, Jun Yin^1^**

^1^School of Information and Control Engineering, China University of Mining and Technology, Xuzhou, 221116, China

*** Correspondence:**Xing Chen

[xingchen@amss.ac.cn](mailto:xingchen@amss.ac.cn)

**Keywords: microRNA; disease; association prediction; matrix factorization; Kronecker regularized least square**

# Supplementary Table

**Supplementary Table 1.** We applied SNMFMDA to prioritize all the candidate miRNA-disease pairs based on all the known miRNA-disease associations in HMDD database as training samples. This prediction result is released for further experimental validation and research.

# The calculation of miRNA functional similarity

In order to calculate the functional similarity between the two miRNAs, it is necessary to consider the diseases associated with these two miRNAs. We need to define the semantic similarity between disease and disease group at first. Here, we use $d$ to indicate one disease and $D=\{d(1),d(2),\ldots d(t)\}$ to represent one disease group containing $t$ diseases. We define the semantic value between the disease $d$ and disease groups $D$ as the maximum value of the semantic similarity between the disease $d$ and all diseases in the disease group $D$. Therefore, the semantic similarity $S(d,D)$ between disease $d$ and disease groups $D$ is calculated as follows:

$$S\left( d,D \right)=\max_{1\leq i\leq m} S(d,d(i))$$

For miRNA $\boldsymbol{m}\boldsymbol{(}\boldsymbol{1}\boldsymbol{)}$, we define the disease group $\boldsymbol{D}\boldsymbol{1}\boldsymbol{=\{}\boldsymbol{d}_{\boldsymbol{1}}\boldsymbol{(}\boldsymbol{1}\boldsymbol{),}\boldsymbol{d}_{\boldsymbol{1}}\boldsymbol{(}\boldsymbol{2}\boldsymbol{),\ldots}\boldsymbol{d}_{\boldsymbol{1}}\boldsymbol{(}\boldsymbol{m}\boldsymbol{)\}}$ whose element $\boldsymbol{d}_{\boldsymbol{1}}\boldsymbol{(}\boldsymbol{i}\boldsymbol{)}$ was associated with miRNA $\boldsymbol{m}\boldsymbol{(}\boldsymbol{1}\boldsymbol{)}$. Similarly, we define another disease group $\boldsymbol{D}\boldsymbol{2}\boldsymbol{=\{}\boldsymbol{d}_{\boldsymbol{2}}\boldsymbol{(}\boldsymbol{1}\boldsymbol{),}\boldsymbol{d}_{\boldsymbol{2}}\boldsymbol{(}\boldsymbol{2}\boldsymbol{),\ldots}\boldsymbol{d}_{\boldsymbol{2}}\boldsymbol{(}\boldsymbol{n}\boldsymbol{)\}}$ for miRNA $\boldsymbol{m}\boldsymbol{(}\boldsymbol{2}\boldsymbol{)}$, and there is known association between disease $\boldsymbol{d}_{\boldsymbol{2}}\boldsymbol{(}\boldsymbol{j}\boldsymbol{)}$ and miRNA $\boldsymbol{m}\boldsymbol{(}\boldsymbol{2}\boldsymbol{)}$. The functional similarity between $\boldsymbol{m}\boldsymbol{(}\boldsymbol{1}\boldsymbol{)}$ and $\boldsymbol{m}\boldsymbol{(}\boldsymbol{2}\boldsymbol{)}$ could be calculated as follow:

$$FS\left( m\left( 1 \right),m\left( 2 \right) \right)=\frac{\sum_{1\leq i\leq m} S(d_{1}\left( i \right),D2)+\sum_{1\leq j\leq n} S(D1,d_{2}(j))}{m+n}$$
